# Comprehensive Analysis of Cardiovascular Diseases: Symptoms, Diagnosis, and AI Innovations

**DOI:** 10.3390/bioengineering11121239

**Published:** 2024-12-07

**Authors:** Muhammad Raheel Khan, Zunaib Maqsood Haider, Jawad Hussain, Farhan Hameed Malik, Irsa Talib, Saad Abdullah

**Affiliations:** 1 Department of Electrical Engineering, The Islamia University of Bahawalpur, Bahawalpur 63100, Pakistan; engr.mraheelkhan@gmail.com; 2 Department of Biomedical Engineering, Riphah College of Science and Technology, Riphah International University, Islamabad 46000, Pakistan; jawad.hussain@engineer.com; 3 Department of Electromechanical Engineering, Abu Dhabi Polytechnic, Abu Dhabi 13232, United Arab Emirates; 4 Mechanical Engineering Department, University of Management and Technology, Lahore 45000, Pakistan; irsa.talib@umt.edu.pk; 5 School of Innovation, Design and Engineering, Division of Intelligent Future Technologies, Mälardalens University, 721 23 Västerås, Sweden

**Keywords:** cardiovascular disease, electrocardiography, artificial intelligence, diagnostic methods, machine learning, deep learning

## Abstract

Cardiovascular diseases are some of the underlying reasons contributing to the relentless rise in mortality rates across the globe. In this regard, there is a genuine need to integrate advanced technologies into the medical realm to detect such diseases accurately. Moreover, numerous academic studies have been published using AI-based methodologies because of their enhanced accuracy in detecting heart conditions. This research extensively delineates the different heart conditions, e.g., coronary artery disease, arrhythmia, atherosclerosis, mitral valve prolapse/mitral regurgitation, and myocardial infarction, and their underlying reasons and symptoms and subsequently introduces AI-based detection methodologies for precisely classifying such diseases. The review shows that the incorporation of artificial intelligence in detecting heart diseases exhibits enhanced accuracies along with a plethora of other benefits, like improved diagnostic accuracy, early detection and prevention, reduction in diagnostic errors, faster diagnosis, personalized treatment schedules, optimized monitoring and predictive analysis, improved efficiency, and scalability. Furthermore, the review also indicates the conspicuous disparities between the results generated by previous algorithms and the latest ones, paving the way for medical researchers to ascertain the accuracy of these results through comparative analysis with the practical conditions of patients. In conclusion, AI in heart disease detection holds paramount significance and transformative potential to greatly enhance patient outcomes, mitigate healthcare expenditure, and amplify the speed of diagnosis.

## 1. Introduction

Cardiovascular diseases (CVDs) fatally impact human beings. They are deemed to be one of the leading factors behind incessantly rising morality rates worldwide, contributing to nearly 31% of global deaths—with a stark increment from 17.9 million in 2019 to 18.6 million in 2023 [1]. In this regard, the sheer severity and spread of such diseases compel countries to earmark a considerable amount to expedite the process of early diagnosis and optimized treatments to preempt dire ramifications effectively and, simultaneously, to provide adequate health facilities to the patients [2].

Moreover, in a significant observation by [3], a profound proportion of the estimated costs annually spent on CVDs could be avoided by introducing resourceful preventive techniques, early detection and treatment methodologies, and effective posttreatment management. Furthermore, several risk factors and underlying conditions associated with CVDs could be controlled and managed by promptly identifying the severity of the diseases and, subsequently, adopting a balanced diet, engaging in regular physical activity, and effectively managing stress [4,5].

On the other hand, identifying and diagnosing CVDs presents a significant challenge in contemporary healthcare, as these ailments fall under a broader category with frequently overlapping symptoms across different conditions within the group [6,7]. Conventional diagnostic methods for CVDs incorporate essential ECG, imaging techniques, and clinical examination, but they are considerably prone to errors and misinterpretations—as researchers indicate that one-third of ECG interpretations exhibit significant mistakes, raising concerns regarding the accuracy and reliability of human ECG assessments in clinical practice [8,9]. However, in recent years, the integration of machine learning (ML) techniques has revolutionized the field of medical diagnostics, offering promising solutions for early detection and accurate risk assessment [10,11,12]. ML models excel at processing and extracting meaningful patterns from vast amounts of data, allowing for more accurate and personalized diagnoses [13,14]. Subsequently, ML algorithms optimally collate and analyze the data to detect and classify various CVDs, such as arrhythmias, myocardial infarction, and heart failure [15,16,17]. Integrating multiple data sources, such as medical imaging, genetic data, and patient demographics, can further enhance the accuracy of diagnosis by considering a comprehensive set of features [18]. Moreover, by training these models on large datasets encompassing diverse patient populations, the algorithms could learn to recognize complex relationships and make accurate predictions. However, it is essential to note that machine learning models should be validated and tested rigorously before being integrated into clinical practice because accurate interpretation of these models is critical to gaining the trust of healthcare professionals. Hence, by leveraging the power of advanced algorithms and vast amounts of patient data, ML algorithms can enhance our ability to identify, predict, and prevent cardiovascular diseases [19,20].

AI has the potential to revolutionize cardiovascular healthcare. By synthesizing various patient data, such as genetic profiles, lifestyle factors, and imaging results, AI can create individualized treatment plans, leading to customized therapeutic interventions [21,22].

Furthermore, the integration of AI with remote monitoring technologies and wearable devices has expanded the scope of cardiac healthcare, enabling continuous real-time monitoring beyond clinical environments. These techniques hold great promise in improving early detection, precision medicine, and, ultimately, patient outcomes in the field of cardiovascular health [23,24]. While the integration of AI in cardiology renders hopeful improvements, it also presents complex hurdles, for example, ethical considerations, algorithmic biases, data security, and regulatory compliance—a few of the challenges that need to be addressed. Therefore, the efficient integration of AI tools into current healthcare systems is a critical topic that requires thorough analysis and the development of creative solutions [19,25,26,27,28].

In this article, we will explore the symptoms and risk factors associated with CVDs, examine the existing methods used for detection, and delve into the promising role of artificial intelligence algorithms in enhancing early detection and prediction of the disease. Combining medical knowledge with advanced data analysis techniques can pave the way for a future where CVDs are detected earlier, treated more effectively, and lives are saved. The article is structured so that we first discuss CVDs and provide background information on the diseases, their symptoms, and the existing methods for their detection. Then, we discuss the application of machine learning in the early detection of cardiovascular diseases (CVDs) and which algorithms are effective for different data types.

Each section of the paper focuses on different aspects of cardiovascular diseases and the use of AI-based techniques in their diagnosis and management. Firstly, the research highlights the significance of artificial intelligence in the medical sciences and delineates the types of cardiovascular diseases, their symptoms, and diagnostic methods. Afterwards, the research comprehensively evaluates the resourcefulness of multiple AI, ML, and deep learning (DL) techniques and the role of AI in supporting diagnosis processes. Moreover, a comprehensive discussion of AI algorithms and their applications, particularly emphasizing the role of electrocardiograms (ECGs) in predicting heart conditions, is also presented in this research. Conclusively, the research underscores the future scope and challenges in the field by summarizing the key insights and findings.

## 2. Cardiovascular Disease Background and Classification

**Figure 1:**
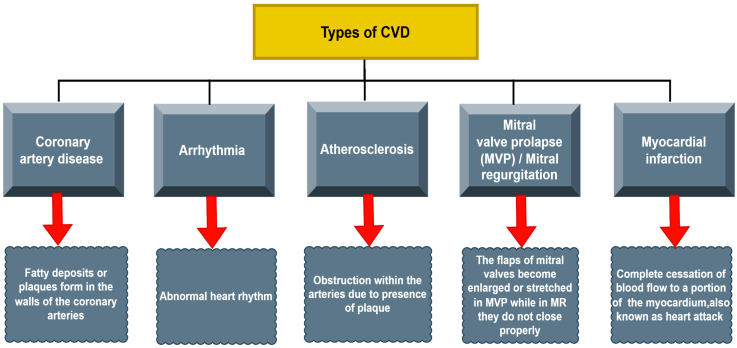
Different CVD conditions discussed in the article.

**Figure 2:**
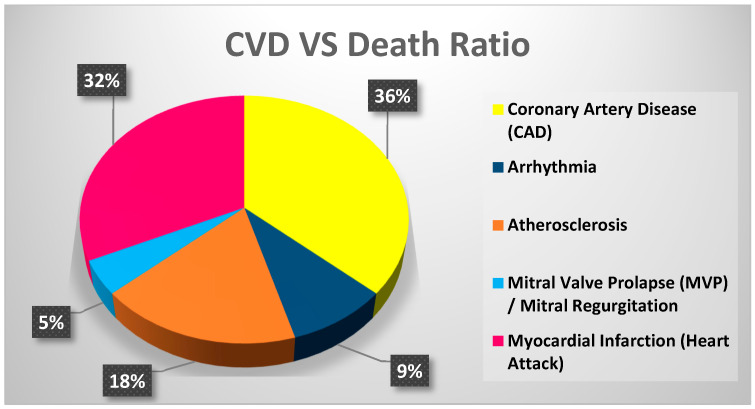
Estimated casualties by different CVDs. (Stats taken from https://professional.heart.org/ accessed on 13 September 2024).

CVDs are a class of diseases that affect the heart and blood vessels [6]. They include conditions such as coronary artery disease, heart attacks, stroke, heart failure, hypertension, valvular heart disease, aortic aneurysm, deep vein thrombosis (DVT), arrhythmias, atherosclerosis, and peripheral artery disease as depicted in Figure 1. These diseases are often caused by genetic, environmental, and lifestyle factors and account for many casualties worldwide, as depicted in Figure 2 [29,30]. In this regard, significant symptoms of CVDs include chest pain or discomfort, fatigue, shortness of breath, irregular heartbeats, dizziness, and swelling, to name a few [31]. Moreover, some non-invasive methods to diagnose these diseases are also thoroughly discussed, including electrocardiography (ECG/EKG), Holter monitoring, echocardiography, Doppler ultrasound, cardiac CT scan, MRI, and stress testing [32]. Notably, the following section provides an in-depth study of five major types of cardiovascular diseases: coronary artery disease, arrhythmia, atherosclerosis, mitral valve prolapses (MVP)/mitral regurgitation, and myocardial infarction. The section discusses each disease’s defining characteristics, underlying causes, and common symptoms.

### 2.1. Coronary Artery Disease: Symptoms and Diagnostic Techniques

CAD, also known as coronary heart disease (CHD) or ischemic heart disease, is when the coronary arteries that supply blood to the heart become narrowed or blocked, usually due to plaque buildup [33]. It primarily affects the coronary arteries, which supply oxygenated blood to the myocardium (heart muscle). It is characterized by atherosclerosis, a process involving the accumulation of cholesterol, fatty deposits (plaques), and inflammatory cells within the arterial walls. These plaques gradually narrow the coronary arteries, reducing the blood flow to the heart [34]. As the coronary arteries narrow, the blood supply to the myocardium becomes inadequate to meet the oxygen and nutrient demands during increased cardiac activity or stress. This disparity between oxygen supply and demand leads to episodes of myocardial ischemia, characterized by chest pain or discomfort, also termed angina pectoris [35].

#### 2.1.1. Symptoms

The development of atherosclerosis in the coronary arteries is caused by hypertension (high blood pressure), dyslipidemia (abnormal lipid profile), diabetes mellitus, smoking, obesity, sedentary lifestyle, and genetic predisposition [36,37].

The first and most alarming symptom of CAD is chest pain, which leads to angina. It can be described as a feeling of pressure, tightness, squeezing, or burning in the chest and may be mistaken for indigestion or heartburn [38]. Additionally, difficulty in breathing is the second most noteworthy symptom of CAD. It becomes more prominent while performing a physical activity or exertion. Moreover, sudden rapid, irregular heartbeats are also a symptom of CAD; fatigue, weakness, or dizziness may indicate reduced blood flow to the heart. In some cases, nausea or vomiting related to chest pain is also observed in heart patients [39].

#### 2.1.2. Diagnostic Techniques for CAD

The choice of diagnostic method depends on various factors, including the patient’s symptoms, medical history, and the healthcare provider’s judgment [40]. A combination of techniques may be used to determine the presence and severity of CAD [41]. Some widely utilized diagnostic procedures include ECG or EKG, which detect electrical signals from the heart that help identify abnormal heart rhythms and detect any signs of inadequate blood flow to the heart muscle [15]. Furthermore, the echocardiogram is another technique to detect CAD, and it utilizes ultrasound waves to create images of the heart. It can provide detailed information about the heart’s function, structure, and blood flow capability [42].

Similarly, a cardiac CT scan is a preferred technique for detecting CAD. Meanwhile, cardiac catheterization and angiography are employed to detect the exact location of the blockage. It involves inserting a catheter and guiding it to the coronary arteries. A contrast dye is injected through the catheter, and X-ray images (angiograms) are taken to visualize the blood flow and any blockages [43]. An advanced technique includes injecting a radioactive substance into the blood and using unique cameras to detect the reduced blood flow areas [44]. A stress test often evaluates the heart’s response to physical activity or induced stress. A medical practitioner observes the outputs of the abovementioned techniques to detect irregularity in the heart. While analyzing the images or signals, human error is always possible. Humans’ situational limitations in analyzing the available data to diagnose a particular pathology may lead to incorrect decisions [45]. For that, computerized detection, based on machine learning and deep learning methods, has been employed to detect the pathology.

### 2.2. Arrhythmia: Symptoms and Diagnostic Techniques

**Figure 3:**
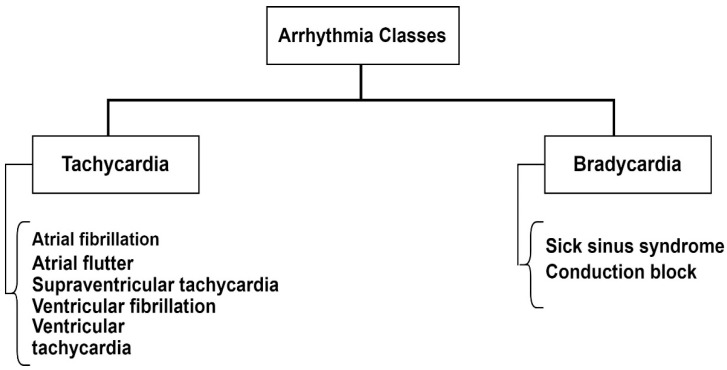
Types of arrhythmia.

Arrhythmia is a medical condition characterized by an abnormal heartbeat [46]. The individual with arrhythmia may suffer from a high heart rate (tachycardia), low heart rate (bradycardia), or irregular heart rhythm [47]. Generally, the heart contracts and relaxes to complete a cycle. This contraction pumps blood to the body with pressure and the relaxation of heart muscles, allowing the deoxygenated blood to reach the heart. Atrial fibrillation, ventricular fibrillation, and atrial flutter are significant arrhythmia types based on the occurrence of irregularity, as depicted in Figure 3.

#### 2.2.1. Symptoms

The irregularity in the electrical signals of an ECG can be due to several reasons. The most important one is the presence of some other heart condition such as CAD, heart attack, cardiomyopathy, etc. [48,49]. In addition to this, abnormal levels or imbalance of electrolytes such as sodium, potassium, calcium, or magnesium may cause disruptions in electrical impulses [50]. An overactive or underactive thyroid gland may also cause arrhythmia.

Furthermore, another alarming symptom is palpitations, which are defined as the sensations of irregular heartbeats, whether fluttering, pounding, or racing. As the heartbeats are irregular, they make the blood flow irregularly to the brain, thus resulting in dizziness or faintness. This may also lead to fatigue and weakness throughout the body, while some patients report chest pains. The arrhythmic patients may also suffer from shortness of breath during physical activities. It should be noted that some arrhythmic patients may be asymptotic and may not demonstrate any symptoms [51].

#### 2.2.2. Diagnostic Techniques for Arrhythmia

Detecting arrhythmia could be challenging as its symptoms may appear irregularly. Further, the heart’s electrical activity must be monitored over an extended period to assess the type, severity, and underlying cause of abnormality in the rhythm [52]. The first technique is ECG, in which the heart’s electrical activity is measured non-invasively using electrodes over the skin. Moreover, stress tests have also been used to identify arrhythmia [53], in which the patient is asked to perform physical activity, and the heart’s activity is monitored using an ECG. Similarly, the Holter monitor may be used for prolonged ECG monitoring continuously for 24–48 h. It may help in detecting intermittent arrhythmia [54]. An event monitor is another device that can be used for up to one month, and the patient activates the device when experiencing symptoms, allowing the doctor to correlate the symptoms with the heart’s rhythm at the time of the event [55]. Electrophysiology study (EPS) is an invasive technique in which flexible wires are threaded through blood vessels into the heart, and these wires help map the heart’s electrical activity. An implantable loop recorder (ILR) is also an invasive technique in which a device is placed under the skin, typically on the chest, to monitor the heart’s electrical activity. The ILR device may remain in the patient for up to 3 years. It helps detect arrhythmias that occur infrequently or in patients with unexplained fainting spells. Cardiac MRI offers high-resolution imaging to assess heart structure and function, particularly in diagnosing arrhythmias with structural heart diseases [56,57,58].

### 2.3. Atherosclerosis: Symptoms and Diagnostic Techniques

Atherosclerosis is a medical condition characterized by the buildup of plaque inside the arteries, which carry oxygen-rich blood to various parts of the body. This plaque, composed of cholesterol, fatty substances, calcium, cellular waste, and other materials, causes the arteries to narrow and harden, restricting blood flow and potentially leading to serious cardiovascular complications [59]. The key difference between atherosclerosis and CAD is that atherosclerosis may occur anywhere in the body, while CAD, as the name suggests, occurs only in the coronary arteries [60]. Due to hypertension, inflammation, tobacco consumption, and high cholesterol levels, the endothelium may become damaged. In response, the body’s immune system tries to repair the damaged portion, which leads to plaque accumulation over time. If the condition is left untreated, it may lead to organ failure or abrupt changes in heart rate patterns [61].

#### 2.3.1. Symptoms for Atherosclerosis

Atherosclerosis in coronary arteries leads to CAD, while it may lead to strokes or transient ischemic attacks when it occurs in carotid and cerebral arteries. In peripheral arteries, the condition may lead to reduced blood flow to the body, which may cause complications and reduced healing capacities of body parts or even gangrene. Due to its similarity to CAD, it also shares the symptoms [62]. The feeling of squeezing or pressure, chest pain that leads to angina, or emotional stress are symptoms that it shares with CAD. It may also cause shortness of breath due to a lack of oxygenated blood to the heart. It may also cause dizziness, severe sudden pain in some parts of the body, loss of control, stroke, or transient ischemic attacks [63].

Several types of atherosclerosis have their own location in various body parts. Atherosclerosis of the coronary arteries leads to the heart and manifests itself in ischemic heart disease, angina pectoris, and myocardial infarction and includes pain and pressure in the chest. A fruitful process affects even the aorta and leads to complications—the aortic wall may tear, producing aortic stressing aneurysms which lead to life-threatening conditions [64].

#### 2.3.2. Diagnostic Techniques for Atherosclerosis

Physical examinations and blood tests are often performed as the first diagnostic step for the detection of atherosclerosis, and patients prone to high blood pressure, high cholesterol levels, and high levels of low-density lipoprotein and who smoke are highly vulnerable to this disease [65]. Moreover, stress tests may also be employed to analyze the areas of the heart with reduced blood supply due to atherosclerosis. ECG, angiography with X-ray, and echocardiography are used extensively for the diagnosis of the disease. Besides these techniques, several other imaging techniques, such as magnetic resonance imaging (MRI), computed tomography angiography, carotid ultrasound, and intravascular ultrasound, have also been employed to see the areas with reduced blood supply and blockages [66]. As mentioned by [67], it is difficult to diagnose atherosclerosis at early stages correctly. However, the development of atherosclerosis does affect gene expression levels before morphological abnormality of the tissue appears. Although there has been significant advancement in imaging techniques over the past few years, the detection of tiny plaques, or the presence of plaques in regions with complex anatomy, is still difficult and tricky [68]. At the same time, patient factors, such as the presence of metallic implants or the presence of obesity, may cause a great deal of difficulty in the diagnosis of atherosclerosis [69]. To overcome these difficulties, medical practitioners may opt for multiple modalities to have a clearer picture of the presence of a blockage and its accurate location.

It has become popular to use new methods of vascular ultrasound, CT and MRI, and molecular imaging to improve the possibility of detecting the early stages of plaque forming and vascular wall inflammation. Techniques for identifying preclinical atherosclerosis have been outlined, including the flow-mediated dilatation (FMD) test, where a 10% change in the brachial artery diameter is accepted as usual. Furthermore, methods for the evaluation of endothelial function and bioassays are conducted in an effort to improve vascular health and reduce the risk of cardiovascular events.

Additionally, imaging of vulnerable plaques, which have the potential for rupture and subsequent thrombus formation, may culminate in acute coronary syndromes. Vulnerable plaques, particularly those with fibrous caps less than 65 microns thick, have been shown to have increased chances of rupture. These imaging structures are, however, necessary for monitoring the course of anti-atherosclerosis therapy as well as in the phases of development and regression of the plaque dispersion [70,71].

### 2.4. Mitral Valve Prolapse (MVP) and Mitral Regurgitation: Symptoms and Diagnostic Techniques

MVP and mitral regurgitation are conditions affecting the mitral valve, which regulates blood flow between the heart’s left atrium and left ventricle. In MVP, the valve leaflets do not close properly during contraction, leading to bulging back into the left atrium. This can result in mitral regurgitation, where blood leaks backwards into the atrium, affecting heart function [72]. Furthermore, MVP is commonly associated with several thoracic skeletal abnormalities, including pectus excavatum, pectus carinatum, scoliosis, straight-back syndrome, and Marfan syndrome [73]. On the other hand, mitral regurgitation is when the mitral valve cannot close properly, causing blood to flow back into the left atrium during the systole phase. This causes a downgrade in efficiency of the heart and causes fatigue, shortness of breath, and fluid retention [74]. One of the leading known causes of MVP is genetic predisposition. Other causes include connective tissue disorders, such as Marfan syndrome and Ehlers–Danlos syndrome [75].

Moreover, one significant non-auscultatory characteristic of mitral valve prolapse is thoracic skeletal abnormalities. A single connective tissue abnormality during the embryonic development of the atrioventricular valves and the bony thoracic cage may cause the relationship between TSA and MVP. Even if asymptomatic, all TSA patients should have non-invasive tests to check for MVP. The identification of MVP in TSA patients may help prevent cardiac arrhythmia and potentially fatal endocarditis [76].

#### 2.4.1. Symptoms for Mitral Valve Prolapse (MVP) and Mitral Regurgitation

The primary and most prominent symptom of the disease is heart palpitations, i.e., the sensation of sudden irregular heartbeats. These are intermittent and become pronounced during stress, anxiety, or physical exertion. This may cause anxiety and panic attacks [74]. It also causes feelings of squeezing or stabbing in the chest for smaller intervals. However, this particular pain is not solely related to MVP; other pathologies may also be responsible for such pains [75]. Furthermore, as the blood flow is disturbed due to mitral valve dysfunction, the patient may suffer from shortness of breath, dizziness or lightheadedness, and fatigue. In addition, some patients also experience migraines or headaches due to MVP. On the other hand, peripheral edema is also a symptom that usually appears in mitral regurgitation in the legs, ankles, and feet. In this condition, the heart does not pump blood effectively, accumulating fluids in the body and causing swelling in different parts [77].

The symptoms of mitral regurgitation include palpitations, shortness of breath, especially in exertion or a supine position, and chest pain. In severe cases, patients may have fatigue and weakness due to decreased cardiac output and other fluid-related symptoms such as swelling in the legs, ankles, or feet, a persistent cough, and, at times, pink frothy sputum. In extreme situations, vertigo, hypotonia, or syncope with potential heart failure symptoms is possible. In contrast, visible symptoms of accumulation of fluids, transitory thrombosis of veins of the upper or inferior extremities, jugular distension, and enlarged liver may manifest. This condition, if not well treated, may result in more severe conditions ranging from pulmonary hypertension to more serious heart failure [78].

#### 2.4.2. Diagnostic Techniques for Mitral Valve Prolapse (MVP) and Mitral Regurgitation

Usually, patients with MVP demonstrate a distinctive “click” sound, followed by a murmuring sound that can be heard using a stethoscope. These sounds are typical of MVP, while murmurs may also be caused by mitral regurgitation [74]. An echocardiogram is another method to detect MVP and mitral regurgitation; a transesophageal echocardiogram (TEE) is sometimes used. In TEE, a small ultrasound probe is inserted into the body through the esophagus, giving a more precise picture of the heart, especially the mitral valve. Cardiac MRI is also used to detect the conditions [79,80]. In addition to these imaging techniques, ECG, Holter monitors, and stress tests have also been used to detect the conditions. In the case of ECG, the electrical activity of mitral valve dysfunction affects the signals coming to the leads attached to the inferior side [81]. MVP causes an inversion of T-waves in those regions and this has been taken as a diagnostic test for the presence of MVP in the general public [82].

The diagnosis and the prediction of the severity for the condition of mitral regurgitation (MR) cannot be made without an echocardiography examination. Generally, transthoracic echocardiography (TTE), which is the most common method used, measures the degree of MR by the regurgitant jet area and the width of the vena contractor according to echocardiographic MRef and volume, and MR is severe when RVol ≥ 60 mL, RF ≥ 50%, or VC width > 0.7 cm. Where there is a need for anatomical structures that determine the image, transesophageal echocardiography (TEE) is primarily helpful by providing high resolution and for operation-planning stages. In contrast, 3D echocardiography improves accuracy in the evaluation of valve shape. Where echocardiography is not helpful or inconclusive, CMR is useful as a second option to measure RVol, RF, and LV volumes, where Rvol > 50 mL predicts increased risk of mortality and surgery in asymptomatic patients with MR. Four-dimensional flow MRI and aggraminium late nuclear resonance imaging are used to measure abdominal blood and provide a quantitative assessment of the impact of fiber tissue, which is needed to ascertain the which is needed to ascertain the likely course of the event for the MR condition [83,84].

### 2.5. Myocardial Infarction: Symptoms and Diagnostic Techniques

Myocardial infarction (MI) is a condition that is caused by the blockage of arteries that supply oxygenated blood to the heart muscles. MI causes the death of heart cells in areas deprived of oxygenated blood. The critical difference between CAD and MI is that CAD refers to the narrowing or blockage of coronary arteries. In contrast, MI is the complete blockage of oxygenated blood to the heart. MI is a more acute manifestation of CAD, but not all cases of CAD result in MI [85]. CAD is a chronic condition characterized by the narrowing of arteries. At the same time, MI refers to a sudden event due to the complete blockage of fresh blood to a portion of the heart, which leads to the death of that particular portion.

#### 2.5.1. Symptoms for Myocardial Infarction

As it is a sudden condition, the symptoms of MI are immediate and must be treated as a priority. The first symptom of MI is chest pain and discomfort, which travels to other areas, such as the arms, shoulder, jaws, back, and neck. The pain occurs along with cold sweats and shortness of breath. Nausea and vomiting are also significant symptoms of MI that occur along with chest pain [86]. Some people also experience dizziness and lightheadedness that may lead to fainting. These symptoms are due to a lack of proper blood supply to the heart, which cannot perform its regular duties [87].

#### 2.5.2. Diagnostic Techniques for Myocardial Infarction

Medical practitioners usually use ECG graphs as the first identifiers for myocardial infarction. The ST-segment depression or elevation symbolizes a heart attack or MI. Stress tests and Holter monitors are also used to identify MI [17]. Different imaging techniques, such as echocardiography, nuclear imaging, coronary angiography, computed tomography angiography, and MRI, have been used extensively to identify heart attack or myocardial infarction [88]. In addition to these non-invasive techniques, a cardiac enzyme test is also used to determine the number of different enzymes and proteins in the blood when a portion of the heart is damaged. The most common marker is troponin, which is not usually found in the blood. It is present in the heart muscles and is leaked to the bloodstream when the heart muscles are damaged [89].

## 3. Artificial Intelligence and Cardiovascular Diseases

Artificial intelligence (AI) means creating machines that simulate human intelligence and can learn and make decisions themselves. It refers to the processes best represented by artificial neural networks that employ data and algorithms to complete tasks such as speech recognition, image analysis, problem solving or prediction, and many others with minimum or no human interaction. It is used in healthcare, finance, transport, and entertainment sectors to change industries by automating operations and improving productivity

### 3.1. AI Applications in Cardiovascular Diseases: Diagnosis and Treatment

There has been a significant amount of research employing machine learning techniques to predict CVDs using non-invasive signals [32,90]. In the following sections, we will explore different CVDs, their most common symptoms, non-invasive techniques used to detect their presence, and machine learning algorithms/models that have been employed to detect them. Special emphasis has been given to efficiency of classification during clinical trials.

**Figure 4:**
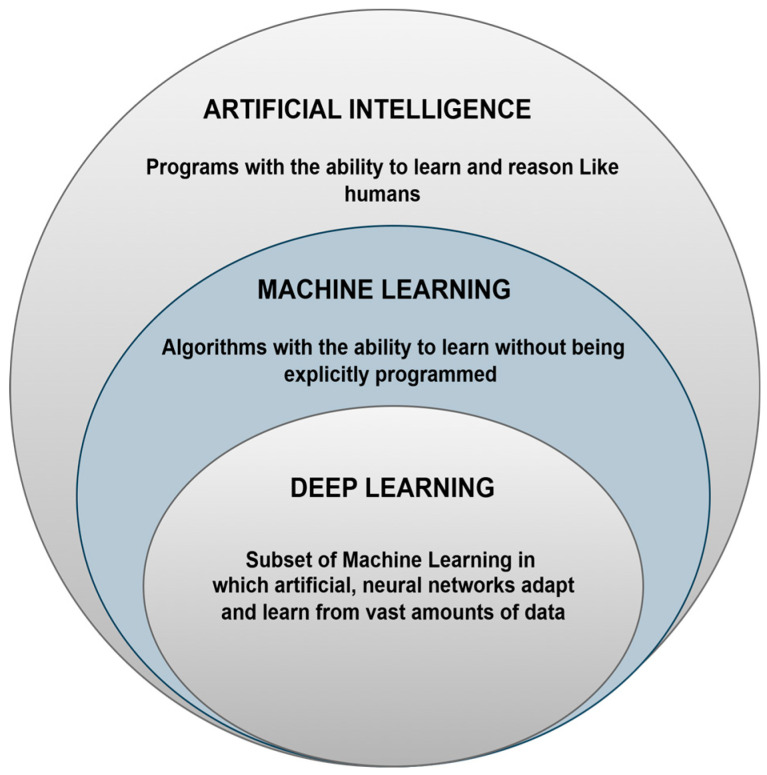
Relationship between AI, ML, and DL.

Artificial intelligence (AI) can potentially advance the diagnosis and treatment of cardiovascular diseases. AI algorithms developed through multi-modal imaging, including MRI, CT, and echocardiograph, have proven helpful in diagnosis. These algorithms are particularly effective in analyzing large-scale healthcare databases to identify and evaluate heart diseases such as heart failure and coronary artery disease. As much as prediction models based on AI techniques provide better results than more conventional methods, challenges lie in data representativeness and incorporation into clinical use. Computing systems with self-learning logic that can reason, learn, and solve problems create AI similar to humans’ cognitive level. Primary applications include vision, speech, decision making, and translation. ML is a subtask that generates predictive models from data; a DNN utilizes deep learning techniques to autoanalyze data trends that lead to increased productivity and decision making across various sectors [27,91,92,93]. The AI hierarchy is shown in Figure 4.

The early-stage and effective detection of cardiovascular diseases (CVDs) can be seen in the application of several machine learning (ML) systems, such as support vector machines (SVMs), artificial neural networks (ANNs), and logistic regression, which achieved about 90% prediction accuracy in diseases such as coronary artery disease and heart failure. The methodology embraces collecting the clinical data, with the data cleaning requirement and the data division, into a training set and testing set for the practical training and evaluation of the models deployed [94].

Multi-modal machine learning (ML) algorithms are suitable for tackling diverse forms of data such as text, audio, pictures, and sensor data in diagnosing and predicting cardiovascular diseases [95]. Moreover, convolutional neural networks (CNNs) are alternatively used to interpret images and ECG. At the same time, ECG readings are recurrently fed into recurrent neural networks (RNNs) for pattern recognition, and support vector machines (SVMs) help in the texture analysis of cardiac imaging. Random forest (RF) focuses on risk prediction, especially in hypertension. Apart from these processes, data collection from clinical documents, for example, electronic health records, to create a populated database also employs natural language processing (NLP) and disease detection processes leveraging CAD systems that analyze medical images for diseases such as coronary artery disease. Furthermore, to diagnose and predict cardiovascular disease (CVD) using wearable sensor data, multiple AI techniques like SVMs, decision trees, CNNs, and LSTMs have also been employed [96,97,98].

Some models often employ a technique known as ensembling, which combines multiple base models to optimize the predictive performance of CVD [99]. On the other hand, different approaches are used in ECG data processing and CVD (arrhythmia) identification, like principal component analysis (PCA) for the changes in morphology, wavelet-based algorithms for arrhythmia detection, and k-NN for the classification of the beats. For signal enhancement and QRS detection, an empirical mode decomposition (EMD) approach is used [100,101,102].

**Figure 5:**
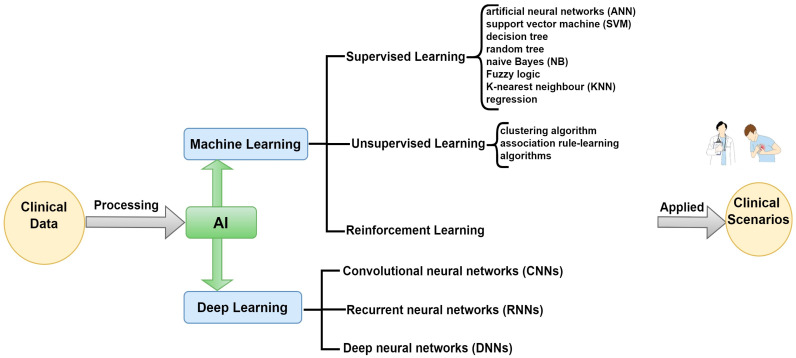
Process of AI in clinical practice. (Reproduced from article https://doi.org/10.1186/s40001-023-01065-y) (accessed on 12 November 2024) [92].

Hybrid methods combining different DL approaches, such as CNNs with BiLSTMs or SNNs, enhance the detection and classification of ECG abnormalities [103]. Furthermore, spiking neural networks (SNNs), which mimic the human brain by transferring information through spike signals, offer energy-efficient solutions for real-time ECG monitoring in ultra-low-power wearable devices [104]. Additionally, a CNN using 12-lead ECG data demonstrated potential as a diagnostic tool for hypertrophic cardiomyopathy (HCM) after further validation. GaborCNN models have been used to classify various cardiovascular diseases, reducing computational complexity compared to standard CNN models [105]. Figure 5 shows the learning process for ML and DL algorithms. ML methods which use feature extraction that relies on ground information about physiology have been widely used in the diagnosis and prognosis of heart diseases.

**Figure 6:**
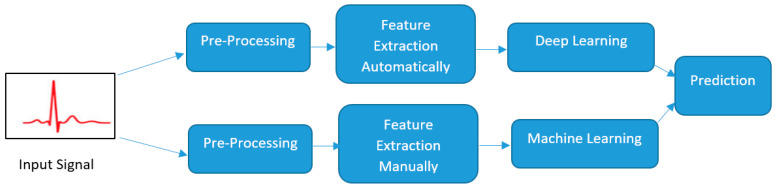
AI Tools used for Cardiovascular Disease Diagnosis/Prediction, Especially for ECG.

Thus, AI can contribute to a more efficient and improved diagnostic process in relation to CVD. Software methods such as modern machine learning and deep learning techniques have provided higher accuracy in recognizing images of various organs, including the heart, from echocardiograms, CT scans, and MRI. Compared to manual diagnoses, where human eyes may miss some subtle signs of conditions like CAD and MI, these technologies can be a big help through diagnostic and therapeutic interventions. Furthermore, ECG signals can be evaluated with the required accuracy using AI algorithms, identifying critical arrhythmias and other significant heart disorders requiring emerging treatments, as represented in Figure 6. Compared to the classical machine learning approach, deep learning approaches can autonomously learn features from raw data when they are used for detecting cardiac structural or functional information or proactive prediction [91,106,107].

The literature revealed a number of AI algorithms being used for classification and detection of different CVD conditions. Table 1 provides an overview of various cardiovascular diseases, the AI, ML, and DL algorithms applied to each condition, and their respective applications in detection, diagnosis, monitoring, and personalized treatment planning.

Table 2 summarizes various studies on these diseases, including details of data types, AI methodologies used, and their respective outcomes and implications. The table also highlights the key observations from each study and discusses potential implications or future directions for research, offering insights into the effectiveness and limitations of AI methods in diagnosing and managing cardiovascular conditions.

#### 3.1.1. AI-Based Diagnosis of CAD

Convolutional neural networks (CNNs) are among the most employed methods to detect CAD, and several researchers have achieved promising results. The authors of [123] analyzed over 20,000 angiograms using DNN models and achieved an accuracy of 98.4% to recognize the vessel segment and an F1 score of 0.829 for stenotic lesion detection. The authors of [124] used ECG signals for the classification of regular and CAD patients by employing a CNN and achieved an accuracy of 98.97%, sensitivity of 98.87%, and specificity of 99.01%. The results seem ideal, but they are achieved in a controlled environment. Another study [125] decomposed the ECG signals and performed R-peak detection before feeding into a CNN. It achieved an accuracy of 80.10%, with 81% sensitivity and 79.3% specificity.

#### 3.1.2. AI-Based Diagnosis of Arrhythmia

Yong Xia et al. [126] focused on proving performance during inter-patient analysis. The researchers used a lightweight transformer combined with a CNN and a denoising autoencoder. They used two scenarios, one with a trained autoencoder and the other without training and achieved accuracies of 97.93% and 97.66%, respectively. Pławiak et al. [127] used a deep genetic ensemble of classifiers (DGEC) on the MIT-BIH arrhythmia database to achieve a sensitivity of 94.62%, specificity of 99.66%, and accuracy of 99.37%. The developed algorithm is efficient enough to work on mobile devices efficiently and provide results immediately. Moreover, Yang Weiyi et al. [128] focused on heartbeat noise in ECG signals and used a principal component analysis network (PCANet) along with a linear support vector machine (SVM) to increase the speed of the classification process. They claimed 97.77% and 97.08% accuracy for skewed and noisy heartbeat and ECG data.

To successfully identify arrhythmias using ECG signals, the most widely used model is a convolutional neural network (CNN) with an 84–99.6% accuracy on various datasets. The specificity of these models, which shows how many arrhythmia cases are detected as such, ranges from 68.29% to 96.19%. It should also be noted that while these methods report high rates of diagnostic accuracy, there is a very significant drop in performance when more complicated inter-patient comparisons are undertaken [138]. In addition, CNN, SVM, and LSTM were applied for ECG data obtained from wearable devices; the accuracy of arrhythmia detection was reported to be as high as 99.84%, similar to coronary artery disease and myocardial infarction with values such as 99.26% by using a custom single-lead ECG device [139]. However, in specific cases, certain models have seen noticeable improvements. For instance, convolutional neural network (CNN) deep learning models achieved 100% accuracy in several studies, while 85–98% accuracy was reported across different datasets [140].

#### 3.1.3. AI-Based Diagnosis of Atherosclerosis

Due to the difficulty of recognizing the blockage’s presence and correct locality, scientists have applied different machine learning methods to detect and diagnose the disease efficiently. Pend et al. [67] carried out genetic classification of healthy and atherosclerosis patients at early stages. Three ML algorithms were used (support vector machine (SVM), random forest (RF), and linear regression (LR)), and accuracies of 75.58%, 63.57%, and 63.95%, respectively, were achieved. Data were taken from an online database, namely, the Gene Expression Omnibus database. Chen et al. [129] used hospital data to classify regular patients using random forest and ensemble learning. The purpose of using different algorithms was to use all features considered in this study. The authors used the area under the receiver operating characteristic (AUC) curve as the efficiency measurement factor of the algorithm. RF provided a result of 0.8826, which is increased to 0.896 when ensemble learning is used. When feature optimization techniques were used using the Dijkstra algorithm, the final AUC achieved was 0.9170. The study by Biswas et al. [130] used a two-stage deep learning model to identify and measure plaque size using ultrasound images. The first stage extracts the region of interest, while the other measures the plaque size. The authors achieved an error of 0.0935 ± 0.0637 mm for carotid intima-media thickness and 2.7939 ± 2.3702 mm^2^ for plaque area.

#### 3.1.4. AI-Based Diagnosis of MVP and MR

As the condition is not critical, only a handful of studies have explored MVP using AI techniques. Untreated MVP may lead to more severe conditions, such as ventricular arrhythmia [141], ischemic stroke [142], bacterial endocarditis [143], and such; hence, more focus has been given to related conditions. Further, as MVP is more pronounced in older people, studies of MVP in young adults are relatively minimal. Gen-Min Lin et al. [131] used 12-lead ECG signals to predict MVP in young army personnel. The authors used support vector machine (SVM), logistic regression (LR), and multi-layer perceptron (MLP) classifiers to classify patients and healthy controls. The algorithms demonstrate 69.85%, 72.10%, and 70.04% specificities, respectively, and accuracies of 69.93%, 72.10%, and 70.11%, respectively. All three algorithms show a sensitivity of 72.22%.

Tesson et al. [132] predicted MVP in middle-aged and elderly people using a gradient boost machine (GBM) classifier. The authors used areas under the receiver operating characteristic curve to predict diseases and found 77% for MVP. The authors mentioned that predicting MVP from ECG signals is the most challenging as it minimally affects ECG morphology. The top predictor of MVP is PR duration, which is the early portion of QRS from lead V2 and V3.

Chest radiographs have been used by Daiju Ueda [133] to diagnose mitral regurgitation using a CNN-based deep learning AI model. The accuracy, sensitivity, and specificity of the model were 73%, 71%, and 74%, respectively. In another study, Feifei Yang et al. [134] developed a self-supervised learning (SSL) algorithm that can help physicians diagnose the severity of mitral regurgitation using color video Doppler echocardiography. The authors claimed that the model increased the sensitivity from 77% to 86.7% when the algorithm outputs were provided to physicians, while the specificity remained unchanged (91.5% to 90.5%).

Several methods have recently been utilized for MR assessment with the assistance of artificial intelligence and have proved to be valuable solutions. Among the techniques applied to Doppler echocardiography using deep learning methods, the diagnostic sensitivity was approximately 90%. Regarding specificity, the 89% to 93% range was achieved for classification issues related to severe MR using the CNN model [144]. The total functional segment used has a U-Net with a dice similarity score of 0.85-0.92, which means the accuracy of the segmentation is high. The other result indicated that automated echocardiographic analysis decreased time by over 50% [145]. The linear-regression-based models for EROA quantification also reported a robust correlation with ground truth values, with all correlations being over 0.9 [146]. Models that can reconstruct flow did well in terms of accuracy, having an error margin of about five to fourteen per cent. Predictive accuracy rates of 85% in hybrid clinical decision support systems were attained. One study reported that the prognostic models that predict adverse events have consistently improved outcome prediction correctness by as much as 20% [147].

#### 3.1.5. AI-Based Diagnosis of Myocardial Infarction

Being an acute condition, the most critical parameter is time to detect and diagnose the myocardial infarction. Using the troponin test, James McCord et al. [135] took age, sex, and cardiac troponin at 0 and 30 min after the patients arrived at the hospital. The authors divided the patients into three groups, low risk, intermediate risk, and high risk, and monitored the patients after 30 and 45 days. The machine learning algorithm accurately predicts the major adverse cardiac events in high-risk patients. The algorithm provides 100% accuracy. In another study, Jia-Zheng Jian et al. [136] tried to solve the problem of overfitting and underfitting in convolutional neural networks to detect MI in ECG signals. The authors developed two algorithms (multi-lead features-concatenate narrow network and multi-scale features-concatenate networks) and achieved accuracies of 95.76% and 61.82%. The authors mentioned that the issue of imbalance in class and quality of extracted features is yet to be explored in detail.

In another interesting study by Xiaoqin Song et al. [137], gene data were investigated to determine the differences between MI patients and healthy humans. The authors used SVM and achieved an accuracy of 87%. Another study by Li-Ming Tseng et al. [148] used ECG signals to locate the occluded coronary artery in patients with ST-elevation myocardial infarction. The authors used short-time Fourier transform (STFT) and continuous wavelet transform (CWT) for signal processing, which was then sent to a CNN for classification. STFT achieved an accuracy of 79.3%, while with CWT, it is 83.7%. The specificity and sensitivity of prediction depend on the presence of occlusion.

## 4. Discussion

This article reviews five different heart conditions, their symptoms, and typical diagnostic techniques/methods and AI methods utilized to classify these heart conditions.

ECG is the most used data type/technique and is the first test to diagnose heart conditions. As it is related to the pumping of the heart, any change in the pumping pattern affects the electrical signals. Sinoatrial (SA) specialized cell nodes originate these electrical signals, which are then spread through upper chambers, causing them to pump blood to lower chambers (ventricles). These electrical signals then travel through the atrioventricular (AV) node, which delays the propagation of these signals for some time, allowing the ventricles to fill with blood. As the ventricles are filled with blood, the electrical signals travel through the lower part of the heart, causing it to pump, thus spreading the blood throughout the body. Any change in this workflow causes abnormality in the heart, which is visible through ECG [149]. Features of ECG are summarized in Table 3.

Most heart conditions affect some of these segments, and hence the condition may be detected. Sometimes, the same segment is disturbed by different conditions. As an example, a change in ST-segment may refer to acute MI, but at the same time, it may also be due to acute pericarditis and early repolarization patterns [137]. A highly trained and experienced medical practitioner is required to analyze such ECGs. But AI methods have been found to perform the work similar to or better than a well-experienced medical practitioner [165]. In the context of computer-aided diagnosis, the following novel research comprehensively evaluates the utilization of artificial intelligence for identifying and describing the characteristics of symptomatic carotid plaques, a significant cause of ischemic stroke. It is centered on the application of AI in combination with other techniques, including ultrasound (US), computed tomography angiography (CTA), and magnetic resonance imaging (MRI). Moreover, machine learning and deep learning enhance diagnostic performance since they automate the process of plaque segmentation, grading, and prediction of strokes [166].

Another problem found in the literature is using biased parameters for a particular study. Researchers usually use a set of parameters in ML algorithms for classification. Some parameters are inherent properties of a process. For example, the amplitude of surface electromyography (sEMG) and mechanomyography always increases, and the mean frequency continuously decreases with the onset of fatigue [167,168,169]. These two parameters may be used to determine the onset of fatigue, but there may be other reasons for the parameter change. Fatigue also appears in many diseases and all CVDs; thus, using it as a parameter to classify patients and non-patients is not a good selection. As another example, ref. [170] classified healthy and CAD patients using heart rate variability and achieved an accuracy of 96.36% and a respective sensitivity and specificity of 89.74% and 100%. While it is a fundamental fact that cardiovascular diseases affect the heart rate, variability in heart rate is not solely dependent on CVDs. However, another example is using heart rate to distinguish between bradycardia and tachycardia, representing slow and fast heart pumping, respectively [171]. Thus, there is a dire need to select proper parameters containing information related to the disease/condition.

**Figure 7:**
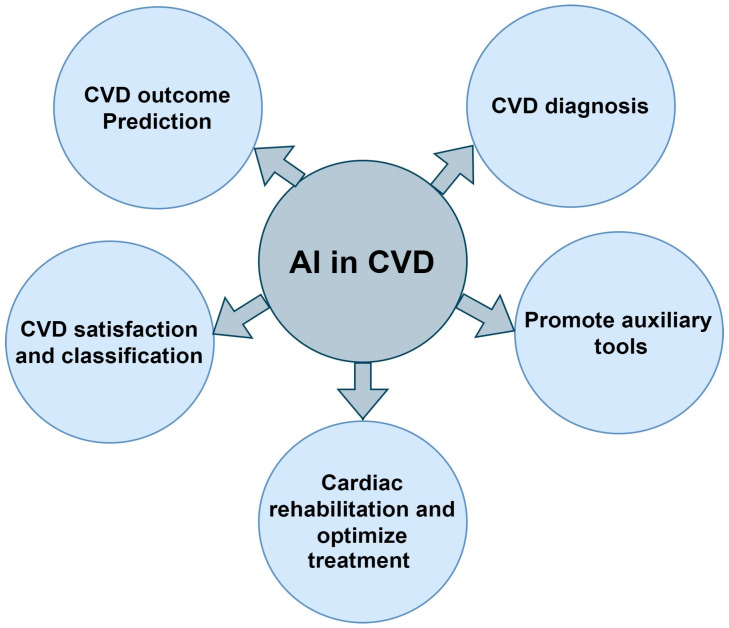
Progress of AI in CVDs (reproduced by paper https://doi.org/10.1186/s40001-023-01065-y [92]).

Imaging techniques such as echocardiography, nuclear imaging, coronary angiography, computed tomography angiography, and MRI have also been used extensively to detect CVD using AI algorithms as represented in Figure 7. The critical issue with these techniques is that they require sophisticated machinery, highly trained staff to handle the machinery, and proper care of the patients, as exposing the patients to radiation is always risky. Also, AI algorithms that use digital images as inputs require high computing power, thus making it difficult to embed the algorithm in computationally limited devices, such as mobile phones [172]. The denoising process of images is also computationally expensive and requires advanced hardware and software. Some researchers have tried to develop computationally feasible algorithms for image processing, but this is still an open area [173]. It has also been observed that most studies used binary classification, disease/no disease, or distinguish between two diseases. This is very useful but has shallow practical utility as real-life scenarios are very different and people having more than one condition are common.

Using deep learning algorithms provides high accuracy and excellent results, but such techniques demand high computation power. Such models may be good and promising but are unrealistic in clinical settings. AI methods, such as a Holter monitor, cardiac event recorder, event monitor, and during-exercise stress test, are most useful in long-term monitoring. There is a chance that a human being (typically a healthcare professional) might miss some crucial slots while observing the extended ECG graphs, either live or recorded. This is not possible with AI-based techniques [174]. 

Sound signals from a stethoscope or Doppler ultrasound have been used extensively to detect heart conditions. The S1 and S2 sounds, marked as the closing of mitral and tricuspid valves and aortic and pulmonary valves, depict the start and end of the cardiac cycle [175]. Phonocardiography has been used to help medical practitioners analyze any abnormality in heart sounds to trace the condition [176]. Some heart conditions may lead to an accumulation of edema and may cause swelling in different parts of the body. If the throat or tongue is affected, speech signals may change, which can give information about the heart condition. Congestive heart failure causes persistent cough and thus may affect speech signals [177]. Such techniques are yet to be explored as only a few studies hypothesized that speech signals may be used to detect heart conditions. 

## 5. Future Scope and Challenges

The above studies show that the role of AI, ML, and DL in diagnosing cardiovascular diseases presents a significant change in healthcare systems. It is equally important to note that the above benefits would also drastically improve disease diagnostics. Patient information such as volume medical imaging (VMI), electronic health records (EHRs), and genomic data can be extensively collected and mined using high-accuracy AI algorithms to identify CVD patterns. Additionally, artificial intelligence can enhance knowledge of individual risks, adjust treatment models and treatments, and consequently enhance patients’ enrollment. Through this feature, predictive models can consider genetic, lifestyle, and clinical data to help with prevention and management, making treatments even more effective and sustainable in terms of the use of available resources.

Their performance also holds promise in other areas, such as real-time and precise CVD surveillance. Wearable devices and mobile health applications can incorporate sensors and autointelligence to follow important markers and identify initial manifestations of cardiovascular calamities that will help healthcare professionals act promptly. Therefore, predictive analysis shows that with the help of advanced technology like AI, physicians or other healthcare providers can monitor risks in advance to enable adequate patient management.

AI can enhance the flow of diagnostic tasks and the overall decision-making processes. The EHR information, both prescribed and implicit, can be efficiently adapted by AI algorithms to understand patient histories, make meaning, and justify clinical decisions. This kind of integration can help boost productivity, decrease the chances of errors made by the technicians, and even increase the quality of the care offered.

While this could offer promising possibilities for improved patient care and potential reductions in healthcare costs, numerous issues, including data credibility, interpretability, clinical implementation, legal treatments, cost issues, and the general stability of RDI models, need to be overcome. Over the years, AI has improved in various fields, including cardiology, but its clinical application remains limited due to unaddressed challenges. One major problem is the AI output, which clinicians may deem a “black box” with many decisions not having clear reasoning paths to explain how clinicians came to certain conclusions. Furthermore, other AI models cannot avoid cross-training class bias, which causes them to misdiagnose people from underdeveloped demographic areas. Another ethical issue is patient confidentiality. Since AI technologies need to process many sensitive patient challenges, the potential of AI in cardiology seems to be at the utmost. Emerging frameworks suggest considerable effort is being directed towards covert, interpretative AI models and securing sensitive datasets. Also, AI, in accompaniment with wearable devices, has a competitive edge in fast and effective patient tracking and hence diagnosing cardiovascular illnesses in early onset scenarios. Another fast-developing application of AI that is likely to create a paradigm shift is using deep learning to deal with big data for personalized medicine and risk assessment.

## 6. Conclusions

AI techniques have been used to monitor cardiac disorders since the origin of AI methods. Though efficient in lab settings, AI techniques are yet to prove their usability in clinical environments, with noisy signals and on computationally limited devices. Parameters must be selected carefully, as different parameters may exist due to different diseases, and the AI algorithm may claim otherwise. This is particularly important as, most of the time, the research focuses on higher accuracy, specificity, and sensitivity rather than choosing a parameter that provides information about the condition. ECG is the most extensively used technique to predict/diagnose a heart condition due to its non-invasive and passive nature. Imaging techniques are used to obtain precise results and the exact abnormality location within and around the heart complex. Further, there is a need for a tertiary or higher classification method that is capable of classifying multiple heart conditions. Recently, some focus has also been given to sound and speech signals, and studies have shown promising results, but more research is needed in this area.

## Figures and Tables

**Table 1:** AI Algorithm classification with respect to CVD.

Reference	CVD	Algorithms (AI, ML, DL)	Applications
[108,109,110]	Coronary Artery Disease (CAD)	ML: SVM, Random Forest, k-NN. DL: CNN, RNN	- Detection via imaging (CT, MRI) - Risk prediction - Personalized treatment plans
[58,101,111,112]	Arrhythmia	ML: Decision Trees, SVM. DL: LSTM, CNN	- ECG signal classification - Real-time monitoring - Predictive alerts
[113,114,115]	Atherosclerosis	ML: Logistic Regression, Random Forest. DL: CNN, GANs	- Plaque detection in arteries (imaging) - Progression prediction - Risk assessment
[98,116,117]	Mitral Valve Prolapse (MVP)	ML: Random Forest, Gradient Boosting. DL: CNN, RNN	- Echocardiogram analysis - Early diagnosis - Treatment recommendations
[92,118,119]	Mitral Regurgitation (MR)	ML: Logistic Regression, SVM. DL: CNN, Autoencoders	- Severity assessment via imaging - Treatment outcome prediction - Surgical decision support
[120,121,122]	Myocardial Infarction (MI)	ML: XGBoost, Random Forest, k-NN. DL: CNN, RNN	- Early detection from ECG - Risk stratification - Postevent prognosis

**Table 2:** Summary of Studies on Cardiovascular Diseases, AI Algorithms, and Their Applications.

Ref	Diseases	Subjects	Source	Data Type	AI Algorithms	Observations	Implications/Future Directions
[123]	Coronary artery disease	10,073 patients	Hospital collection	Angiograms	Deep neural network	Accuracy 98.4%, sensitivity 85.2%, specificity 99.1%	Data are collected from a single hospital and thus lack generality
[124]	Congested heart failure	15 patients, 58 normal	Beth Israel Deaconess Medical Centre Congestive Heart Failure Database, Fantasia Database, and MIT-BIH Normal Sinus Rhythm Database	ECG	CNN	Accuracy 98.97%, sensitivity 98.87%, specificity 99.01%	More training data may be used to train the model
[125]	Atrial fibrillation, myocardial infarction, congestive heart failure, and normal	NA	NA	ECG	CNN	Accuracy 80.10%, sensitivity 81.0%, specificity 79.3%	The model may be extended to more diseases
[126]	Imbalance inter-patient ECG classification	NA	MIT-BIH dataset	ECG	Lightweight Transformer combined with CNN and a denoising autoencoder	Accuracy 97.66%, accuracy 97.93% with pre-trained autoencoder	Further optimization methods may be tested based on ensemble classifiers
[127]	17 arrhythmia classes	29 patients	MIT-BIH arrhythmia database	ECG	Deep genetic ensemble of classifiers (DGEC)	Accuracy 99.37%, sensitivity 94.62%, specificity 99.66%	
[128]	Arrhythmia	48 groups of MLII and V5 leads	MIT-BIH arrhythmia database	ECG	Principal component analysis and support vector machine	Accuracy 97.77% and 97.08%	The method provides robustness to noisy signals
[67]	Atherosclerosis	NA	Gene Expression Omnibus database	Gene expression	Support vector machine, random forest, and logistic regression	Accuracy 75.58%, 63.57%, and 63.95%	The clinical information was not taken into account
[129]	Atherosclerosis	Patients and healthy controls	Data collected at Affiliated Hospital of Nanjing University of Chinese Medicine	25 features affecting atherosclerosis	Random forest and ensemble learning	88.26% and 89.6%	Feature reduction methods may be used to increase the efficiency
[130]	Atherosclerotic carotid wall detection and plaque measurements	408 images from 204 patients	Toho University, Japan	Ultrasound images	Deep learning models	Error of 0.0935 ± 0.0637 mm for carotid intima-media thickness and 2.7939 ± 2.3702 mm^2^ for plaque area	The AI methods demonstrate promising results with patch technique
[131]	Mitral valve prolapse	2206 young male military personnel	Hualien Armed Forces General Hospital, Taiwan	ECG	Support vector machine, logistic regression, and multi-layer perceptron classifiers	Accuracy 69.93%, 72.10%, and 70.11% Specificity 69.85%, 72.10%, and 70.04% Sensitivity 72.22% for all	Feature selection and reduction may be improved in future studies
[132]	Mitral valve prolapse	36,186 ECGs	University of California, San Francisco database	ECG	Gradient boost machine classifier	Receiver operating characteristic curve of 77%	MVP has very limited effect on ECG morphology
[133]	Mitral regurgitation	10,367 radiographs from 7555 echocardiograms in 5270 patients	Osaka City University Hospital, Osaka, Japan	Chest radiographs	CNN-based deep learning	Accuracy 73%, sensitivity 71% and specificity 74%	The accuracy may be increased if left lateral images are taken
[134]	Severity of mitral regurgitation	2766 consecutive echocardiographic studies of patients with MR diagnosed	Chinese PLA General Hospital	Color video Doppler echocardiography	Self-supervised learning (SSL) algorithm	Sensitivity 86.7%, specificity 90.5%	The study provides quantitative indexes to clinicians that assist in diagnosis
[135]	Myocardial infarction	529 patients	Urban emergency department	Age, sex, and cardiac troponin within 0 to 30 min after patient arrival at the hospital	Myocardial ischemic injury index (MI3)	Accuracy 100% for high-risk patients	Other conditions were not considered
[136]	Myocardial infarction	148 patients and 52 healthy controls	PTB ECG database from PhysioNet	ECG	Multi-lead features-concatenate narrow network and multi-scale features-concatenate networks	Accuracy of 95.76% and 61.82%	Imbalance in class and quality of extracted features are yet to be explored
[137]	Myocardial infarction	49 MI patients, 48 normal samples	RNAseq data from the GEO database	Genetic data	SVM	Accuracy 87%	The method is high cost as compared to protein spectrum and serological detection methods

NA = Not available

**Table 3:** ECG features in different cardiovascular conditions.

Reference	CVD	ECG Abnormalities	Symptoms
[150]	Coronary Artery Disease (CAD)	ST-segment depression or elevation, T-wave inversion, pathological Q waves	Chest pain, shortness of breath, fatigue
[151,152]	Arrhythmia	Abnormal P-waves, PR interval, QRS complex, irregular heart rhythms, tachycardia, or bradycardia	Palpitations, dizziness, fainting, shortness of breath
[150,153,154]	Atherosclerosis	Typically no direct ECG abnormalities, secondary effects like arrhythmias if associated with CAD	Chest pain (angina), leg pain during walking, shortness of breath
[155,156]	Mitral Valve Prolapse (MVP)	Inverted or biphasic T-waves in the inferior leads	Palpitations, chest pain, dizziness, fatigue
[157]	Mitral Regurgitation (MR)	Left atrial enlargement (P mitrale), left ventricular hypertrophy (LVH), atrial fibrillation	Shortness of breath, fatigue, palpitations
[158]	Myocardial Infarction (MI)	ST-segment elevation or depression, T-wave inversion, pathological Q-waves	Severe chest pain, sweating, nausea, shortness of breath
[159,160]	Heart Failure (HF)	Low-voltage QRS in peripheral leads, left bundle branch block (LBBB), right bundle branch block (RBBB), atrial fibrillation	Fatigue, leg swelling, shortness of breath, rapid weight gain
[161,162]	Hypertension	Left ventricular hypertrophy (LVH), ST-segment and T-wave changes (strain pattern), prolonged QT interval	Headaches, shortness of breath, nosebleeds, fatigue
[163]	Stroke	ST-segment and T-wave abnormalities in acute settings, prolonged QT interval	Sudden weakness or numbness, confusion, trouble speaking (cardiovascular manifestation)
[151,164]	Cardiomyopathy	Low-voltage QRS, abnormal Q-waves	Shortness of breath, fatigue, swelling in legs, arrhythmias
